# *In situ* observation of macroscopic phase separation in cobalt hexacyanoferrate film

**DOI:** 10.1038/srep42694

**Published:** 2017-02-16

**Authors:** Masamitsu Takachi, Yutaka Moritomo

**Affiliations:** 1 Graduate School of Pure and Applied Science, University of Tsukuba, Tsukuba 305-8571, Japan; 2 Center for Integrated Research in Fundamental Science and Engineering (CiRfSE), University Tsukuba, Tsukuba 305-8571, Japan; 3 Tsukuba Research Center for Interdisciplinary Materials Sciences (TIMS), University of Tsukuba, Tsukuba 305-8571, Japan; 4 Faculty of Pure and Applied Science, University of Tsukuba, Tsukuba 305-8571, Japan

## Abstract

Lithium-ion secondary batteries (LIBs) store electric energy via Li^+^ deintercalation from cathode materials. The Li^+^ deintercalation frequently drives a first-order phase transition of the cathode material as a result of the Li-ordering or Li-concentration effect and causes a phase separation (PS) into the Li-rich and Li-poor phases. Here, we performed an *in situ* microscopic investigation of the PS dynamics in thin films of cobalt hexacyanoferrate, Li_*x*_Co[Fe(CN)_6_]_0.9_, against Li^+^ deintercalation. The thick film (*d* = 1.5 μm) shows a characteristic macroscopic PS of several tens of μm into the green (Li_1.6_Co[Fe(CN)_6_]_0.9_) and black (Li_.6_Co[Fe(CN)_6_]_0.9_) phases in the *x* range of 1.0 < *x* < 1.6. Reflecting the substrate strain, the thin film (*d* = 0.5 μm) shows no trace of the PS in the entire *x* region. Our observation suggests that the macroscopic PS plays a significant role in the charge/discharge dynamics of the cathode.

The lithium-ion secondary battery (LIB)^1^,^2^ is a widely used energy storage device for consumer electronics, such as smart phones and portable computers, and for electric vehicle power systems. In addition, the LIB is a key technology for multiple clean energy applications. Their energy and power densities are predominantly governed by the cathode materials, which store Li^+^ within their crystal structure. Most commercialized cathode materials, such as LiCoO_2_^3^, LiMn_2_O_4_^4^, and Li(Ni_1/3_Co_1/3_Mn_1/3_)O_2_^5^, form solid solutions over a large Li-concentration range. They do, however, show first-order structural phase transitions because of the Li-ordering or Li-concentration effect. These phase transitions cause phase separation (PS) into Li-rich and Li-poor phases, because the local Li concentration is the order parameter. For the purposes of this discussion, we classify the PS into two types, i.e., microscopic PS within the particle and macroscopic PS between the particles. The microscopic PS is initiated by nucleation of the second phase, whose size is determined by the balance between the free energy gain due to the phase transformation and the strain loss at the interface. In this case, the Li^+^ deintercalation process suffers extra kinetic barriers, such as nucleation and the resultant interface strain within the particle. On the other hand, the macroscopic PS is advantageous for battery performance because particle homogeneity reduces the inner stresses and possible mechanical degradation of the material.

The PS problem is most extensively investigated in LiFePO_4_^6^, which stores Li through a two-phase transformation between FePO_4_ and LiFePO_4_^7^,^8^,^9^,^10^. LiFePO_4_ shows very high rate performance resulting from smaller nanoparticles, doping, and surface coatings^11^, although both the phases show low ionic and electronic conductivities. The transmission electron microscopy (TEM) images of chemically deintercalated Li_1/2_FePO_4_ nanoparticles show clear stripe-type phase boundaries within the particle^12^. This microscopic PS is well reproduced by a phase-field model including the elastic coherency strain^13^. On the other hand, Delmas and coworkers^14^ investigated electrochemically deintercalated Li_*x*_FePO_4_ nanoparticles by means of X-ray diffraction and electron microscopy. They concluded that individual particles are essentially FePO_4_ or LiFePO_4_ (macroscopic PS). They interrupted the observation in terms of the ‘domino-cascade model,’ that is, Li^+^ deintercalation takes place just near the phase boundary because nucleation of a new micro-domain of FePO_4_ in another part of the LiFePO_4_ crystal would require much higher energy. Based on the ab initio density functional calculations of Li_*x*_FePO_4_, Malik *et al*.^15^ proposed that the transformation path to the single phase exists even at a very low overpotential. Thus, the PS problems in LiFePO_4_ are still controversial.

Thin film of cobalt hexacyanoferrate, Li_*x*_Co[Fe(CN)_6_]_0.9_, is another ideal platform to investigate the PS dynamics against Li^+^ deintercalation. In addition, yhe Li_*x*_Co[Fe(CN)_6_]_0.9_ film is a promising cathode material for LIB, showing a discharge capacity of 139 mAh/g and average voltage of 3.6 V^16^. Let us consider the structural correlation between Li_*x*_FePO_4_ and Li_*x*_Co[Fe(CN)_6_]_0.9_. Li_*x*_FePO_4_ is built up of two-dimensional (2D) sheets with [FeO_4_]_n_ formula of corner-sheared FeO_6_ octahedra. These sheets are connected by PO_4_ tetrahedra to make a three-dimensional (3D) skeleton. The Li^+^ deintercalation oxidizes Fe^2+^ to Fe^3+^ and causes significant change in the Fe-O bond length, which leads to the PS. On the other hands, Li_*x*_Co[Fe(CN)_6_]_0.9_ is built up of [Fe(CN)_6_]^4−^ octahedra. Similarly to the case of Li_*x*_FePO_4_, these octahedra are connected by Co^2+^ ions to make a 3D skeleton. The Li^+^ deintercalation oxidizes Co^2+^ to Co^3+^ and causes significant change in the Co-N bond length, which leads the PS into the green (high-*x*) and black (low-*x*) phases at *x*~1.2^16^. The green and black phases show the face-centered cubic structure and are formally expressed as Li_1.6_Co^2+^ [Fe^2+^ (CN)_6_]_0.9_ and Li_0.6_Co^3+^ [Fe^2+^ (CN)_6_]_0.9_, respectively. Importantly, phase transformation into the black phase causes significant volume contraction: lattice constants (*a*) are 1.02 nm in the green phase and 1.00 nm in the black phase. The volume change is ascribed to the oxidization and resultant spin state transition of Co. Actually, the X-ray absorption near-edge structure (XANES) around the Co K-edge^16^ indicates that Co^2+^ and Co^3+^ take the high-spin and low-spin states, respectively. Thus, in both the materials, the Li^+^ deintercalation, and resultant oxidization of the 3D skeletons, causes the cooperative structural distortion and PS.

Here, we performed an *in situ* microscopic investigation of PS dynamics in the Li_*x*_Co[Fe(CN)_6_]_0.9_ films with use of the color difference between the green and black phases against Li^+^ deintercalation. In thick film (*d* = 1.5 μm), we observed a characteristic macroscopic PS of several tens of μm into the original green and secondary black phases below *x* < 1.0. The length scale (several tens of μm) is much larger than the crystal grain size (several hundreds of nm). We, however, observed no trace of the PS in thin film (*d* = 0.5 μm) and ascribed the absence of the PS to the strain due to the substrate.

## *In Situ* Observation of PS Dynamics

**Figure 1:**
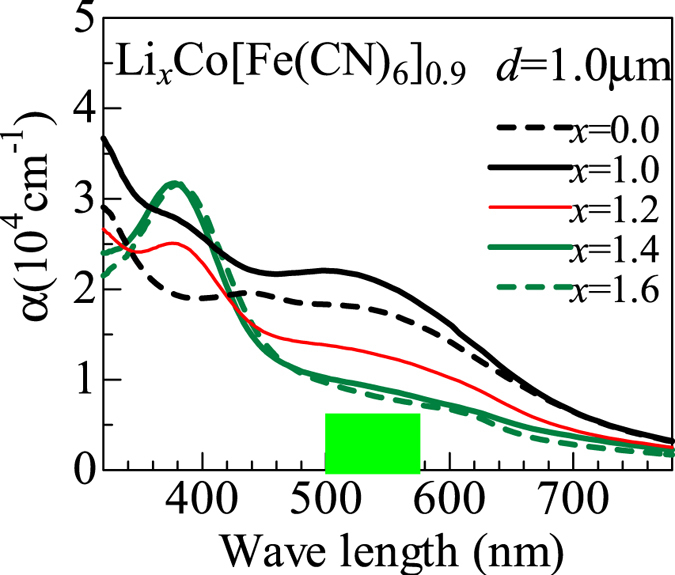
Absorption spectra of Li_*x*_Co[Fe(CN)_6_]_0.9_ film (*d* = 1.0 μm) against *x*. The green square represents the probe light source in the microscopy investigation.

Figure 1 shows absorption spectra of the Li_*x*_Co[Fe(CN)_6_]_0.9_ film against *x*. The film thickness (*d* = 1.0 μm) was chosen so that the minimum transmittance (at 380 nm) becomes ~0.04. The spot of the light source is about 1 mm in diameter. Roughly speaking, the spectra at *x* = 1.6 and 1.0 corresponds to the green and black phases, respectively. In the green phase, the intense absorption observed around 380 nm is ascribed to the electron transfer from Fe^2+^ to the neighboring Co^2+^ 17. In the black phase, the broad absorption observed around 540 nm is ascribed to the electron transfer from Fe^2+^ to the neighboring Co^3+^ 17. The absorption intensity at 540 nm shows significant change in the phase transformation from the green to black phases. So, the 540 nm bands can be used as a sensitive monitor of the respective phases. The perpetration depth at the probe light wavelength is 0.4–1.0 μm. The minimum transmittance at the probe light wavelength is 0.04 even for the thickest (*d* = 1.5 μm) film.

**Figure 2:**
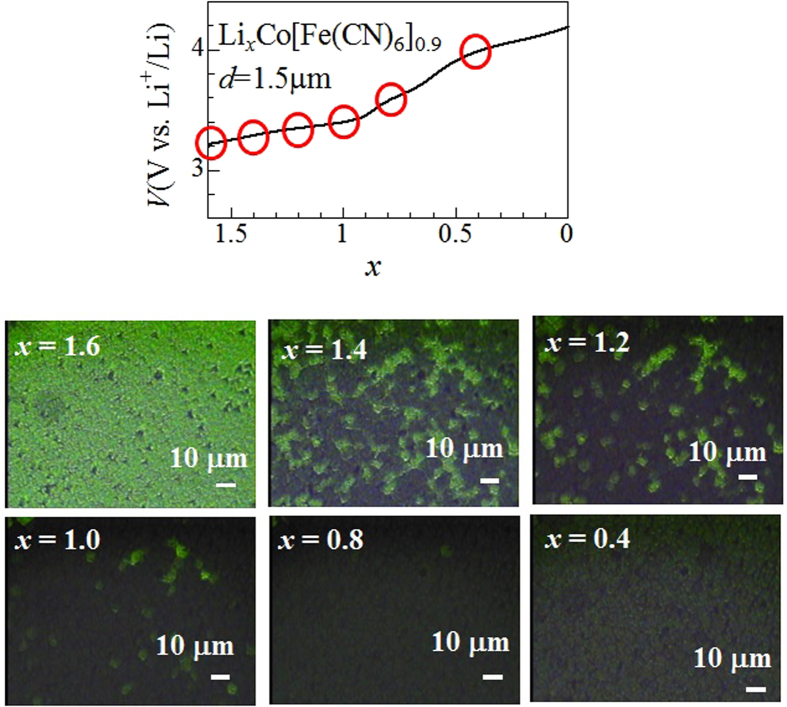
Upper panel: charge curve of Li_*x*_Co[Fe(CN)_6_]_0.9_ film (*d* = 1.5 μm) at 0.7 C against *x*. Lower panel: microscopic images at respective *x*, indicated by red circles in the 1^st^ charge curve. Green and black regions at *x* = 1.0, 1.2, and 1.4 corresponds to the green and black phases, respectively.

**Figure 3:**
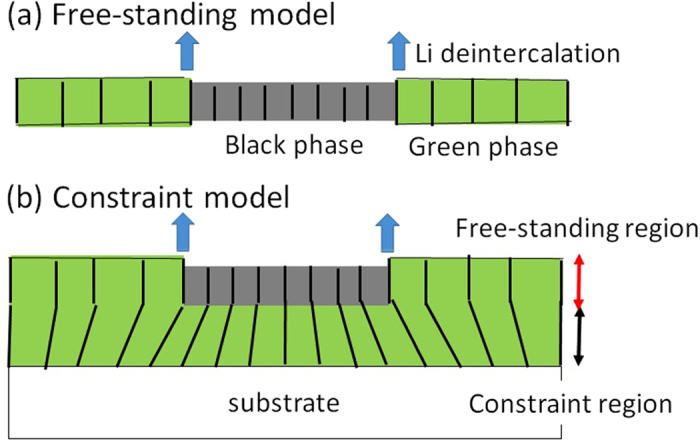
Schematic pictures of macroscopic PS in the (**a**) free-standing and (**b**) constraint models. In the constraint model, the bottom parts of the crystal pillars are strongly pinned at the substrate. Green and gray regions represent green and black phases, respectively. Upper arrows and vertical lines represent the selective Li^+^ deintercalation and crystal grains, respectively.

Figure 2 shows the charge curve of the Li_*x*_Co[Fe(CN)_6_]_0.9_ film (*d* = 1.5 μm) at 0.7 C against *x*, together with the microscopic images. In the late stage (0.6 < *x* < 0.0) of the charge curve, a plateau is observed at around 4.0 V. This plateau is ascribed to the reduction process of Fe^2+^ to Fe^3+^ 16. At *x* = 1.6, the microscopic image is homogeneous and green, indicating that the system is in the green phase (Li_1.6_Co^2+^ [Fe^2+^ (CN)_6_]_0.9_). With decreases in *x*, the black region appears (*x* = 1.4), increases in area (*x* = 1.2 and 1.0), and finally covers the entire image (*x* = 0.8). The black region corresponds to the black phase (Li_0.6_Co^3+^ [Fe^2+^ (CN)_6_]_0.9_) because the region does not transmit the green light (Fig. 1). Thus, we observed macroscopic PS in thick film. We performed Rietveld structural analysis (Rietan-FP^18^) of the synchrotron-radiation X-ray powder diffraction pattern of Li_1.2_Co[Fe(CN)_6_]_0.9_ (Fig. 3S). The *a* values of the green and black phases are 1.01848 ± 0.00006 nm and 0.99535 ± 0.00007 nm, respectively. With further decrease in *x* below *x* = 0.8, the image gradually becomes bright. This is because parts of Fe^2+^, which is the final state of the optical transition, are oxidized to Fe^3+^ with decrease in *x*.

Looking at Fig. 2 (the *x* = 1.4, 1.2, and 1.0 images), the size of the green region gradually shrinks without changing the contrast, indicating that no additional nucleation of the black micro-domain occurs within the green region. That is, the transformation from green (Li_1.6_Co^2+^ [Fe^2+^ (CN)_6_]_0.9_) to black (Li_0.6_Co^3+^ [Fe^2+^ (CN)_6_]_0.9_) phases takes place at the phase boundary via selective Li^+^ deintercalation. We emphasize that the length scale (several tens of μm) of the PS is much longer than that (several hundred nm: see Figs S1 and S2) of the crystal grain size of the film. We consider that the volume contraction due to the phase transformation into the black phase is the main driving force of the macroscopic PS, as schematically shown in Fig. 3(a). In order to confirm this hypothesis, we evaluated the linear expansion coefficient (Δ*L/L*) between *x* = 1.6 and 1.4. The linear expansions (Δ*L*) between *x* = 1.6 and 1.4 were evaluated using the spots at the grain boundaries, as shown in Fig. S5. The Δ*L/L* value is −0.013 ± 0.012 in the black region [Fig. S4(a)] and −0.004 ± 0.013 in the green region [Fig. S4(b)]. The observed Δ*L/L* ( = −0.013) value in the black phase is quantitatively consistent with that ( = −0.023) evaluated from the lattice constants of the green and black phase. In other words, the lattice contraction due to the phase transformation propagates beyond the respective grains. Then, the lattice contraction causes a significant strain at the phase boundary. In such a region, the Li^+^ deintercalation and subsequent phase transformation into the black phase is much easier than nucleation of a new micro-domain of Li_0.6_Co^3+^ [Fe^2+^ (CN)_6_]_0.9_ in another part of the green region. This scenario is essentially the same as the ‘domino-cascade model’ of Li_*x*_FePO_4_^14^.

**Figure 4:**
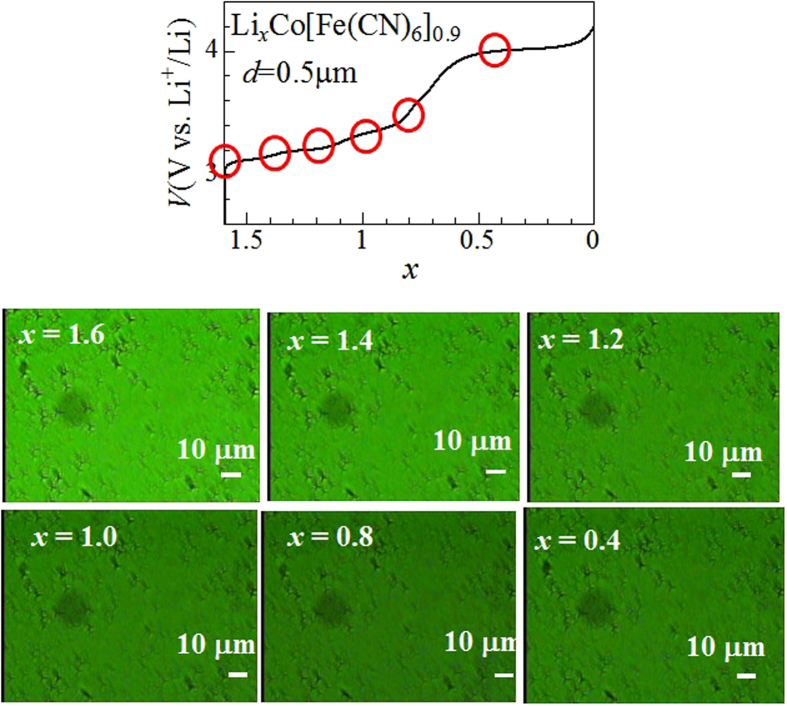
Upper panel: charge curve of Li_*x*_Co[Fe(CN)_6_]_0.9_ film (*d* = 0.5 μm) at 0.9 C against *x*. Lower panel: microscopic images at the respective *x*, indicated by red circles in the charge curve.

The PS dynamics are critically dependent on the film thickness. Figure 4 shows the charge curve of the Li_*x*_Co[Fe(CN)_6_]_0.9_ film (*d* = 0.5 μm) at 0.9 C against *x*, together with the microscopic images. In the late stage (0.6 < *x* < 0.0) of the charge curve, a plateau due to the reduction process of Fe^2+^ to Fe^3+^ is observed at around 4.0 V. We observed no trace of the macroscopic PS. The image becomes dark with a decrease in *x* from *x* = 1.6 to 0.8. This is because parts of Co^2+^ are oxidized to Co^3+^, which is the initial state of the optical transition, with decrease in *x*. With further decrease in *x* below 0.8, the image becomes bright again. This is because parts of Fe^2+^, which is the final state of the optical transition, are oxidized to Fe^3+^ with decrease in *x*.

## Normalized Absorption Intensity *I*_n_ Against *x*

**Figure 5:**
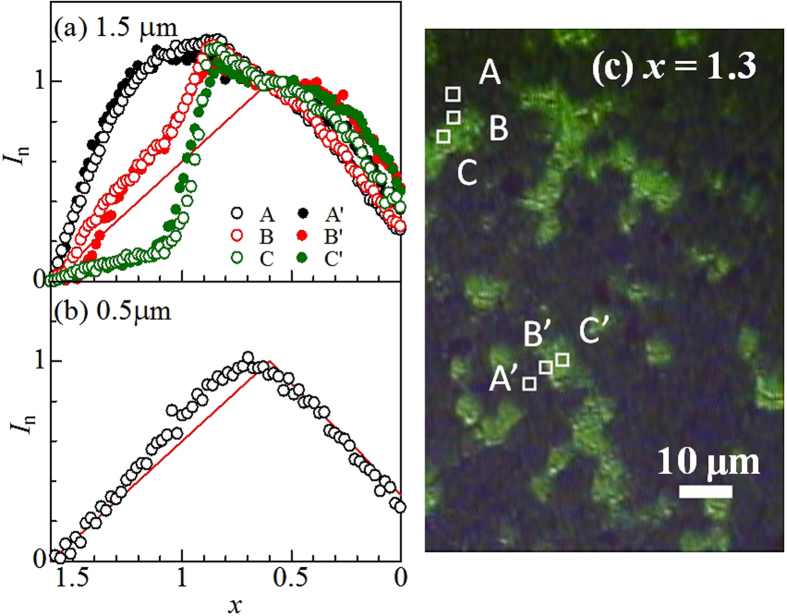
(**a**) Normalized absorption intensity (*I*_n_) of the Li_*x*_Co[Fe(CN)_6_]_0.9_ film against *x*: (**a**) *d* = 1.5 μm at 0.7 C and (**b**) *d* = 0.5 μm at 0.9 C. The red lines are results of the mean-field model (see text). (**c**) Microscopic image of the thick film at *x* = 1.3, together with the investigated positions.

To investigate the PS dynamics in more detail, we quantitatively investigated the absorption intensity against *x*. Recall that the 540 nm absorption band is ascribed to the electron transfer from Fe^2+^ to the neighboring Co^3+^. Then, the absorption intensity at *x* = 1.6 (Li_1.6_Co^2+^ [Fe^2+^ (CN)_6_]_0.9_) and at *x* = 0.6 (Li_0.6_Co^3+^ [Fe^2+^ (CN)_6_]_0.9_) should be a minimum and maximum, respectively. Therefore, we defined normalized absorption intensity (*I*_n_) as [*I(x*) − *I*(1.6)]/[*I*(0.6) − *I*(1.6)]. Here, we assume a homogeneous Co oxidization in the *x* range of 1.6 > *x* > 0.6 and a homogeneous Fe oxidization in the *x* range of 0.6 > *x* > 0.0 (mean-field model). In this model, *I*_n_ is expressed as 1.6 − *x* (1.6 > *x* > 0.6) and *x* + 0.4 (0.6 > *x* > 0.0), because *I*_n_ is proportional to the probability of finding the Co^3+^ site adjacent to the Fe^2+^ site. The red lines in Fig. 5(a) and (b) are the results of the mean-field model.

Figure 5(a) shows *I*_n_ of the thick film against *x* in the black (A and A′), phase boundary (B and B′), and green (C and C′) regions. Data were averaged in 2 × 2 μm^2^ area, as indicated by squares in Fig. 5(c). In the *x* range of 1.6 > *x* > 0.6, the *I*_n_ − *x* curves show significant position dependence and seriously deviate from the mean-field model (red lines). In the *x* range of 0.6 > *x* > 0.0, however, the *I*_n_ − *x* curves overlap each other and nearly obey the mean-field model (red lines). In the black region (A and A′), *I*_n_ steeply increases to ~1 with a decrease in *x* below *x* = 1.2, indicating selective Li^+^ deintercalation and transformation into the black phase. The increase in *I*_n_ gradually saturated below *x* = 1.2, indicating that the black region covers the entire 2 × 2 μm^2^ square. In the green region (C and C′), *I*_n_ remains nearly zero in the *x* range of 1.6 < *x* < 1.0. With further decrease in *x, I*_n_ steeply increases to ~1, indicating that the phase boundary reaches the square. In the boundary region (B and B′), *I*_n_ shows an intermediate behavior between the two limiting cases. The increase of the *I*_n_ − *x* curve, however, is rather gradual. This unexpected behavior implies finite width of the phase boundary due to the gradual change of *x* and/or inclination of the boundary. Figure 5(b) shows the *I*_n_ − *x* curves of the thin film (0.5 μm) at 0.9 C against *x*: The curve nearly obeys the mean-field model (red lines) in the entire *x* region, indicating that Co^2+^ and Fe^2+^ are homogeneously oxidized in the respective plateaus in the thin film.

## Discussion

According to classical nucleation theory, Li deficiencies in the parent Li_1.6_Co[Fe(CN)_6_]_0.9_ pool together to form micro-clusters of Li_0.6_Co[Fe(CN)_6_]_0.9_. The cluster deterministically grows when its size stochastically reaches a critical size, which is determined by the balance between the free energy gain due to the transformation and the strain loss at the interface. Unfortunately, the nucleation process is difficult to detect due to the limited spatial resolution ( = 1 μm) of the optical microscopy. We investigated the microscopic image in the 1^st^ discharge process and found that the black region remains as islands even in the fully discharged state (Li_1.6_Co[Fe(CN)_6_]_0.9_) (the *x* = 1.6 image in Fig. S6). This is in sharp contrast with the initial homogeneous image (the *x* = 1.6 image in Fig. 2) of the ion-exchanged Li_1.6_Co[Fe(CN)_6_]_0.9_. Such islands are probably stabilized by local compression or local Fe deficiency in the film. Thus, physical or chemical inhomogeneity of the cathode materials is advantageous for the PS.

Finally, let us discuss the *d*-dependence of the PS dynamics. If the film were free-standing [Fig. 3(a)] without any constraint, macroscopic PS would be possible even in the thin film. The actual film, however, consists of columnar crystal grains^19^. The bottom parts of the crystal pillars are strongly pinned at the indium tin oxide (ITO) substrate, as schematically shown in Fig. 3(b). We will call this model as “constraint model”. Similarly to the case of the 1.5 μm film, we evaluated Δ*L/L* between *x* = 1.6 and 1.4. The Δ*L/L* value is −0.002 ± 0.006 [Fig. S4(c)]. We observed no detectable displacement in the in-plane direction, which support the constraint model. In the constraint model, the Gibbs free energy change is expressed as ΔG = ΔG_phase transformation_ + ΔG_deformation_. The first term is the Gibbs free energy change due phase transformation in the free-standing system, while the second term deformation energy of the pillars. To realize the PS (ΔG < 0), the energy gain (−ΔG_phase transformation_) due to the phase transformation and the interfacial strain must surpass the energy loss (−ΔG_deformation_) due to the pillar bending. The thicker the film becomes, the smaller −ΔG_deformation_ becomes. Thus, the constraint model well explains why the PS appears in the thick film (Fig. 2) but is absent in the thin films (Fig. 4). Judging from the fact the 1.5 μm film shows the PS while the 0.5 μm film does not, the characteristic thickness is order of ~1 μm. Let us evaluate the bending angle at *d* = 1 μm at the phase boundary. The in-plane displacement is 0.1 μm [=10 μm (domain size of the PS) × 0.01(Δ*L/L* in the black region)]. Then, bending angle becomes 6 degree [=sin^−1^(0.1 μm/1 μm)]. These arguments imply that the external strain due to the surrounding environment crucially influences the PS dynamics within the respective particle, and hence the cycle and rate properties of the cathode. Here, we point out that the cycle properties is much worse in the Li_*x*_Co[Fe(CN)_6_]_0.9_ film^16^ as compared with the isostructural Li_*x*_Mn[Fe(CN)_6_]_0.83_ film^20^, which does not shows PS.

### Summary

We performed *in situ* microscopic observation of PS dynamics in the Li_*x*_Co[Fe(CN)_6_]_0.9_ films. The thick film shows a characteristic macroscopic PS of several tens of μm into the original green and secondary black phases below *x* < 1.0. We further found that the PS is absent in the thin film, reflecting the strain due to the substrate. This suggests that the external strain due to the surrounding environment crucially influences the PS dynamics within the respective particles, and hence the cycle and rate properties of the cathode.

## Method

### Fabrication and characterization of Li_*x*_Co[Fe(CN)_6_]_0.9_ film

Thin films of Li_1.6_Co[Fe(CN)_6_]_0.9_ were synthesized by electrochemical deposition of Na_1.6_Co[Fe(CN)_6_]_0.9_ and successive electrochemical ion-exchange. The electrochemical deposition of the Na_1.6_Co[Fe(CN)_6_]_0.9_ film was performed in a three-pole beaker-type cell. The working, counter, and standard electrodes were an indium tin oxide (ITO) transparent, Pt, and standard Ag/AgCl electrodes, respectively. The electrolyte was an aqueous solution containing 0.8 mmol/L K_3_[Fe(CN)_6_], 0.5 mmol/L Co(NO_3_)_2_, and 5.0 mol/L Na(NO_3_). The films were deposited on the ITO electrode under potentiostatic conditions at −0:45 V vs. the Ag/AgCl electrode. The thickness (*d*) of the film was controlled by the deposition time and was determined with a profilometer. The chemical composition of the film was determined by the inductively coupled plasma (ICP) method and CHN organic elementary analysis (PerkinElmer 2400 CHN Elemental Analyzer). The compound contains crystal waters as Na_1.6_Co[Fe(CN)_6_]_0.9_2.9 H_2_O. Na in the film was electrochemically substituted for Li in a two-pole cell under Ar atmosphere in an Ar filled glove box. The anode was Li and the electrolyte was ethylene carbonate (EC)/diethyl carbonate (DEC) solution containing 1 mol/L LiClO_4_. The charge/discharge rate was about 1 C. The cut-off voltage was from 2.0 to 4.2 V.

The X-ray diffraction patterns of the Na_1.6_Co[Fe(CN)_6_]_0.9_ films were obtained with a Cu Kα lines (Fig. S7). All the reflections can be indexed with the face-centered cubic structure. The lattice constants (*a*) were 1.027 nm for both the films. The morphologies of the Na_1.6_Co[Fe(CN)_6_]_0.9_ films were investigated with atomic force microscopy (AFM: Fig. S1) and scanning electron microscopy (SEM: Fig. S2). The films consist of crystalline grains of several hundred nm in diameter. The cross-sectional SEM image^19^ indicates that the respective crystalline grains are columnar.

### Optical battery cell for microscopy

The optical battery cell has a structure of Li_1.6_Co[Fe(CN)_6_]_0.9_ film on an ITO glass/Teflon sheet with a square hole/anode. The anode was a small piece of Li metal attached on a cupper foil, which was sandwiched between the Teflon sheet and slide glass. The hole in the Teflon sheet was filled with electrolyte. The electrolyte was an ethylene carbonate (EC)/diethyl carbonate (DEC) solution containing 1 mol/L LiClO_4_. The cell was assembled under Ar atmosphere in an Ar-filled glove box and was sealed with Kapton tape. The charge/discharge behavior of the cell was stable and was consistent with the literature^16^ even under air atmosphere for at least ten hours. It is difficult to precisely evaluate the capacity due to the bubbles of Ar gas which were inevitably introduced in the hole. Actually, some parts of the Li_1.6_Co[Fe(CN)_6_]_0.9_ film remained unchanged during the charge/discharge process. Therefore, we assume a fully charged and fully discharged state of *x* = 0.0 and 1.6, respectively.

### *In situ* microscopic observation of the PS dynamics

The *in situ* microscopic PS dynamics were recorded with a microscopy system equipped with a charge-coupled device (CCD) camera for moving images. A halogen lamp was monochromized with a dichroic filter (DIF-50S-GRE: Sigma Koki, Co Ltd.) and used as the probe light source. The transmission range of the filter was 515–560 nm. The spatial resolution of the system was 1 μm. The probe light sensitively monitored the absorption band due to the electron transfer from Fe^2+^ to neighboring Co^3+^. The absorbance intensity *I(x*) at *x* is expressed as −1/*d* × ln[*T(x*)/*T*_0_], where *d, T(x*), and *T*_0_, are the film thickness, transmitted light intensity at *x* and incident light intensity. *T(x*) was evaluated by the chromaticity of the green color. The normalized absorbance intensity (*I*_n_), [*I(x*) − *I*(1.6)]/[*I*(0.6) − *I*(1.6)], is expressed as [ln*T(x*) − ln*T*(1.6)]/[ln*T*(0.6) − ln*T*(1.6)]. To increase the signal-to-noise ratio, the chromaticity was averaged within 8 × 8 pixels (2 × 2 μm^2^ area) around each position.

## Additional Information

**How to cite this article**: Takachi, M. and Moritomo, Y. *In situ* observation of macroscopic phase separation in cobalt hexacyanoferrate film. *Sci. Rep.* **7**, 42694; doi: 10.1038/srep42694 (2017).

**Publisher's note:** Springer Nature remains neutral with regard to jurisdictional claims in published maps and institutional affiliations.

## Supplementary Material

Supplementary Information
