# Supplementary material for: In situ observation of macroscopic phase separation in cobalt hexacyanoferrate film
Source: Sci Rep. 2017 Feb 16;7:42694. doi: 10.1038/srep42694 (PMC5311949; doi:10.1038/srep42694)
Supplement: Supplementary Information [file srep42694-s1.pdf]

Supporting information

In situ observation of macroscopic phase separation in cobalt hexacyanoferrate film

Masamitsu[1] Takachi and Yutaka Moritomo[1-4*]

[1]Graduate School of Pure and Applied Science, University of Tsukuba, Tsukuba 305-8571, Japan

[2]Center for Integrated Research in Fundamental Science and Engineering (CiRfSE), University Tsukuba, Tsukuba 305-8571, Japan

[3]Tsukuba Research Center for Interdisciplinary Materials Sciences (TIMS), University of Tsukuba, Tsukuba 305-8571, Japan

[4]Faculty of Pure and Applied Science, University of Tsukuba, Tsukuba 305-8571, Japan

Contact information:

Yutaka Moritomo

Graduate School of Pure and Applied Science

Center for Integrated Research in Fundamental Science and Engineering (CiRfSE) Tsukuba Research Center for Interdisciplinary Materials Sciences (TIMS)

Faculty of Pure and Applied Science,

Univ. of Tsukuba, Tennodai 1-1-1, Tsukuba 305-8571, Japan

Tel & Fax +81-29-853-4337

e-mail: moritomo.yutaka.gf@u.tsukuba.ac.jp

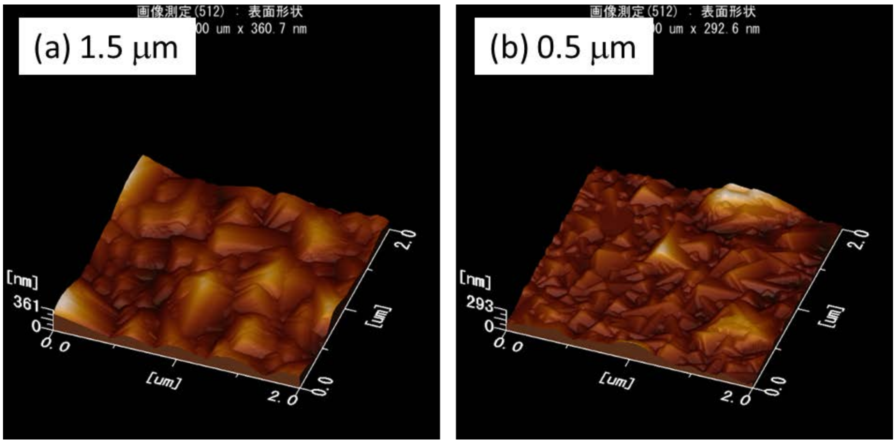

Fig. S1: Atomic force microscope image of $Na_{1.6}Co[Fe(CN)_6]_{0.9}$ film: (a) 1.5 μm and (b) 0.5 μm. Measuremts were performed in the tapping mode. The average raughnesses (*R*a) are 46.6 nm and 34.1 nm for the 1.5 μm and 0.5 μm films, respectively.

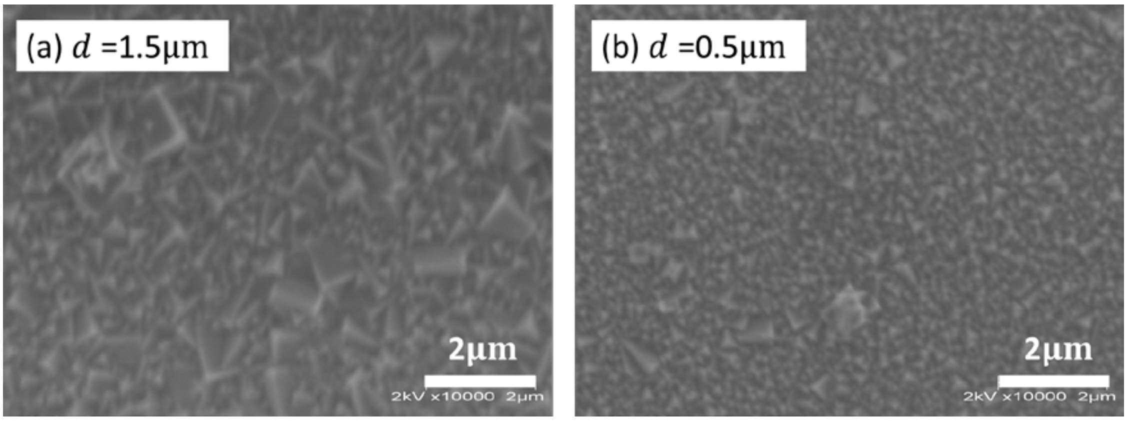

Fig. S2: Scanning electron microscope image of $Na_{1.6}Co[Fe(CN)_6]_{0.9}$ film: (a) 1.5 μm and (b) 0.5 μm. Acceleration voltage was 2 keV.

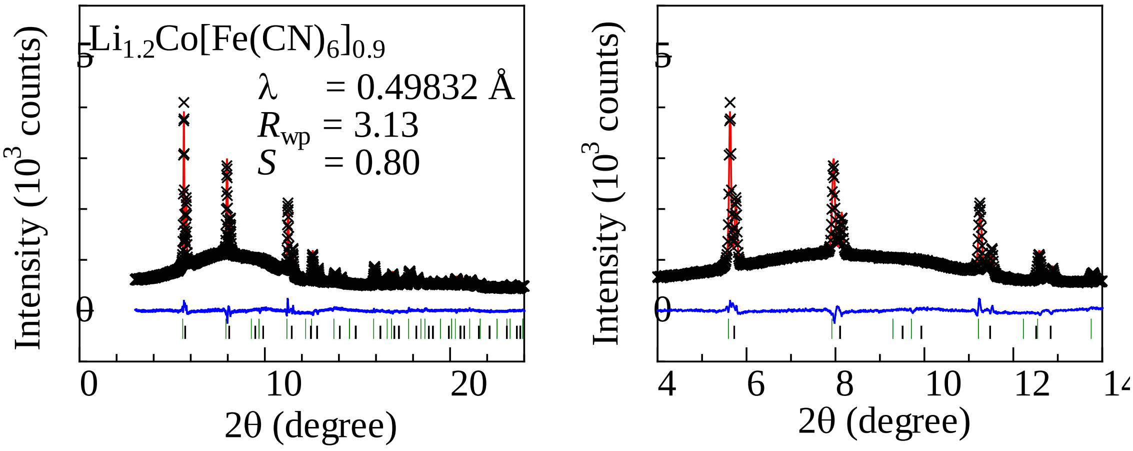

Fig. S3: Left panel: Synchrotron-radiation X-ray powder diffraction pattern (crosses) of the $Na_{1.2}Co[Fe(CN)_6]$ powders peeled from the film. The red curve is result of Rietveld refinement with two face-centered cubic ($Fm\underline{3}m$) phases. The blue curve is the difference between experimental data and calculation. The thin long and thick short bars are the diffraction angles for the green and black phases, respectively. Right panel shows the same patterns with the magnified *x* range.

The synchrotron-radiation X-ray powder diffraction pattern was obtained at BL02B2 beamline of SPring-8. The film was peeled off and was sealed in a 0.3 mmϕ glass capillary. The capillary was placed on a large Debye-Scherrer camera. The wavelength (= 0.049832 nm) of the X-ray was calibrated by the lattice constant of standard CeO_2 powders. The diffraction patterns were detected with an imaging plate.

atom	site	*g*	*x*	*y*	*z*	*B*
Fe	4a	0.9	0	0	0	3.3(2)
Co	4b	1.0	1/2	0	0	3.3
C	24e	0.9	0.201(2)	0	0	3.3
N	24e	0.9	0.299(2)	0	0	3.3
O	32f	0.3625	0.2837(5)	0.2837	0.2837	3.3

Table S1: Atomic coordinates (x, y, z), occupancy (g) and atomic displacement parameters (B) of the green phase of $Li_{1.2}Co[Fe(CN)_6]$ at 300 K. The crystal structure is face-centered cubic ($Fm\underline{3}m$) with a = 1.01848±0.00006 nm. We omitted Li and ligand O in the Rietveld analysis. R_I is 7.97%.

atom	site	*g*	*x*	*y*	*z*	*B*
Fe	4a	0.9	0	0	0	3.7(4)
Co	4b	1.0	1/2	0	0	3.7
C	24e	0.9	0.193(5)	0	0	3.7
N	24e	0.9	0.310(4)	0	0	3.7
O	32f	0.3625	0.210(1)	0.210	0.210	3.7

Table S2: Atomic coordinates (x, y, z), occupancy (g) and atomic displacement parameters (B) of the black phase of $Li_{1.2}Co[Fe(CN)_6]$ at 300 K. The crystal structure is face-centered cubic ($Fm\underline{3}m$) with a = 0.99535±0.00007 nm. We omitted Li and ligand O in the Rietveld analysis. R_I is 6.83%.

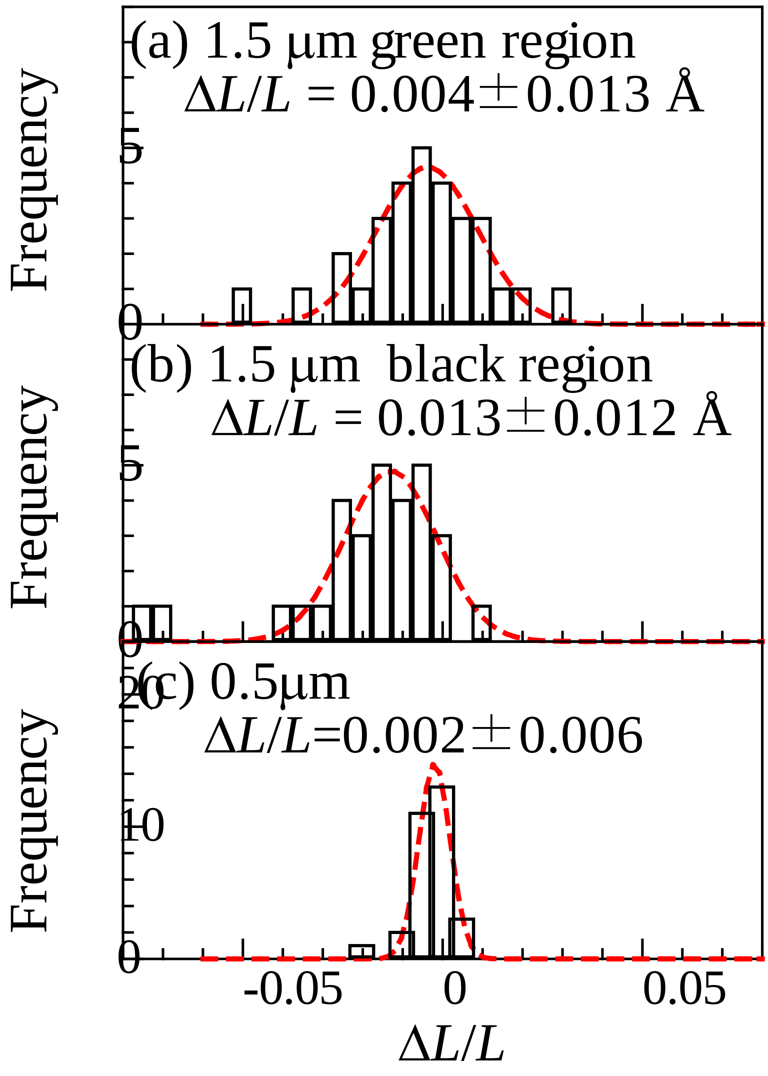

Fig. S4: Distributions of linear expansion coefficient ($\Delta L/L$) between x = 1.6 and 1.4 within (a) green region in 1.5 μm film, (b) black region in 1.5 μm film, and (c) 0.5 μm film. The broken curves represent the results of the Gauss fitting.

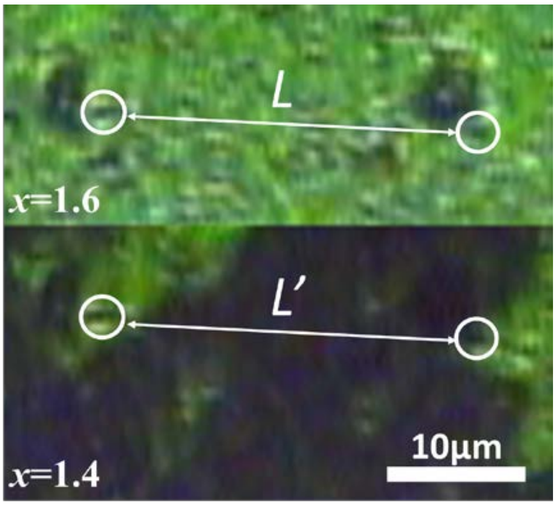

Fig. S5: Explanation how to evaluate linear expansion (ΔL =L'-L). The L values at respective x were evaluated with use of spots at the grain boundaies.

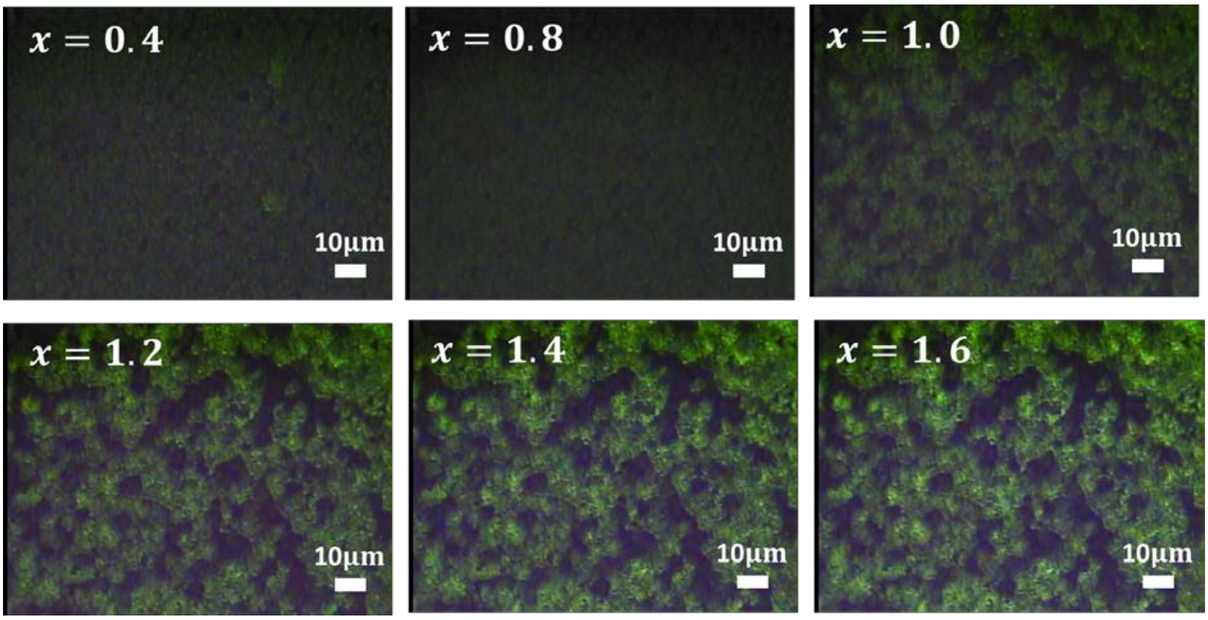

Fig. S6: Microscopic images of $Li_xCo[Fe(CN)_6]_{0.9}$ film (d = 1.5 μm) at 0.7 C at respective x in the 1st discharge process. Green and black regions at x = 1.0, 1.2, and 1.4 corresponds to the green and black phases, respectively.

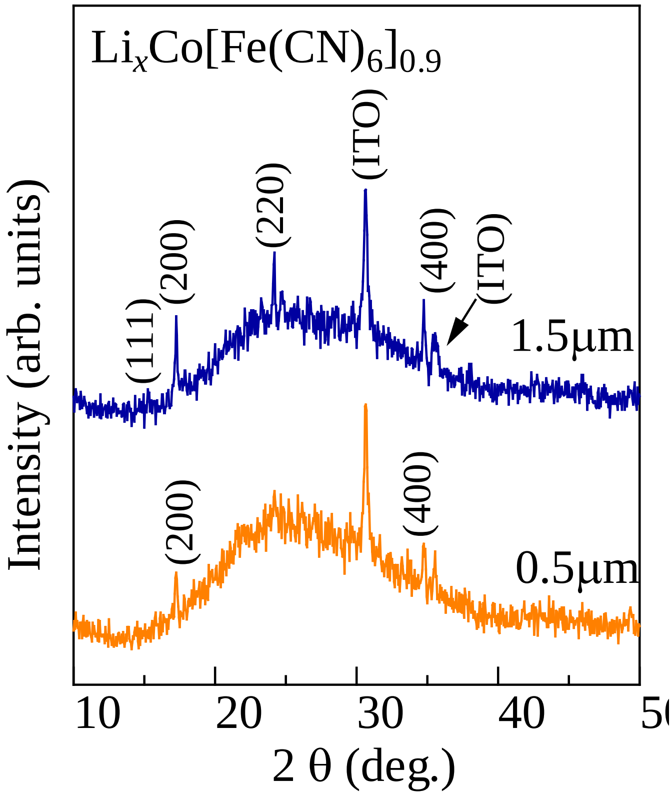

Fig. S7: X-ray diffraction pattern at 300 K for the $Li_{1.6}Co[Fe(CN)_6]_{0.9}$ films with different thickness. The X-ray source was Cu Kα lines. The lattice cosntat (*a*) is 1.027 nm in both the films.
